# First Presentation of Sickle Cell Anemia in A 3.5-Year-Old Girl: A Case Report

**Published:** 2012-03-01

**Authors:** M Pedram, K Jaseb, S Haghi, M Vafaie

**Affiliations:** 1 Thalassemia and Hemoglobinopathy Research Center, Ahvaz Jondishapur University of Medical Sciences, Ahvaz, Iran; 2 Department of Pediatrics Hematology/Oncology, Ahvaz Jondishapur University of Medical Sciences, Ahvaz, Iran

**Keywords:** Sickle cell anemia, Splenic sequestration, Dactylitis

Dear Editor,

Sickle cell disease is an inherited disorder of hemoglobin synthesis, which affects many individuals worldwide. Vaso-occlusive, aplastic and sequestration crises are common in a sickle cell child.[1] One of the leading causes of death in children with sickle cell anemia is an acute splenic sequestration crisis.[2] Children with splenic sequestration disease who have not yet undergone autosplenectomy, as well as older patients with sickle cell disease or S-beta thalassemia, may have sudden, rapid, massive enlargement of the spleen with trapping of a considerable portion of the red cell mass. On physical examination, there may be profound hypotension with cardiac decompensation and massive splenomegaly. The hemoglobin concentration is at least 2 g/dL lower than baseline and is accompanied by a brisk reticulocytosis with increased nucleated red cells and moderate to severe thrombocytopenia.[2] ACS is an acute illness with lung injury characterized by any combination of chest pain, fever, or respiratory symptoms and accompanied by a new pulmonary infiltrate on a chest radiograph.[3] Elevated WBC count, dactylitis, and anemia predicted more severe later outcomes in children younger than 4 years.[4] Dactylitis, often referred to as hand-foot syndrome, is frequently the 1st manifestation of pain in children with sickle cell anemia, occurring in 50% of children by 2 years of age.[5]

**Fig. 1:**
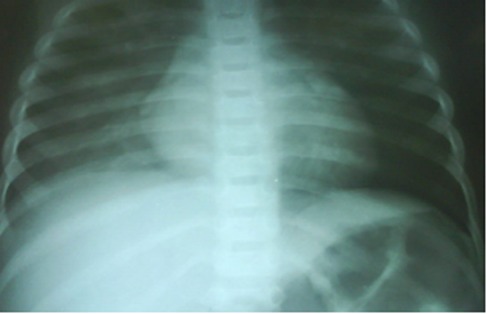
Chest x ray.

A 3.5-year-old girl from race of Arab reffered to Shafa Hospital with severe anemia, thrombocytopenia, leucocytosis and elevated ESR and LDH. Her parents assigned fever, cough, pallor, weakness and tachypnea from six day ago (Figure 1).

Past medical history and familial history of the patient was negative. On physical examination, she had fever with temperature of 39.5°C, severe pallor, pulse rate of 110 per minute, respiratory rate of 32 per minute and blood pressure of 90/60 mmHg. On abdominal examination, she had hepatosplenomegaly. Rales and rhonchi were in both lung fields on respiratory examination while the other systemic examination was essentially normal. Initial laboratory investigations demonstrated hemoglobin of 4.5 gm/dL, white cell count of 19,000/mm(3) (55% neutrophils, 45% lymphocyte), platelet count of 70,000/mm(3) and an erythrocyte sedimentation rate of 45 mm/hour. Renal function tests and urinalysis were normal. A chest radiograph revealed bilateral haziness. For decline of malignancy, bone marrow aspiration was done and discussed reactive bone marrow due to infection.

**Fig. 2:**
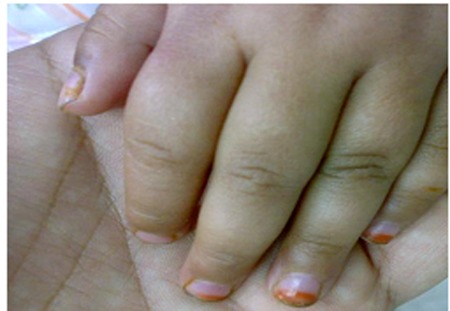
Dactylitis.

After one day a dactylitis was present in hands of the patient (Figure 2).

**Fig. 3:**
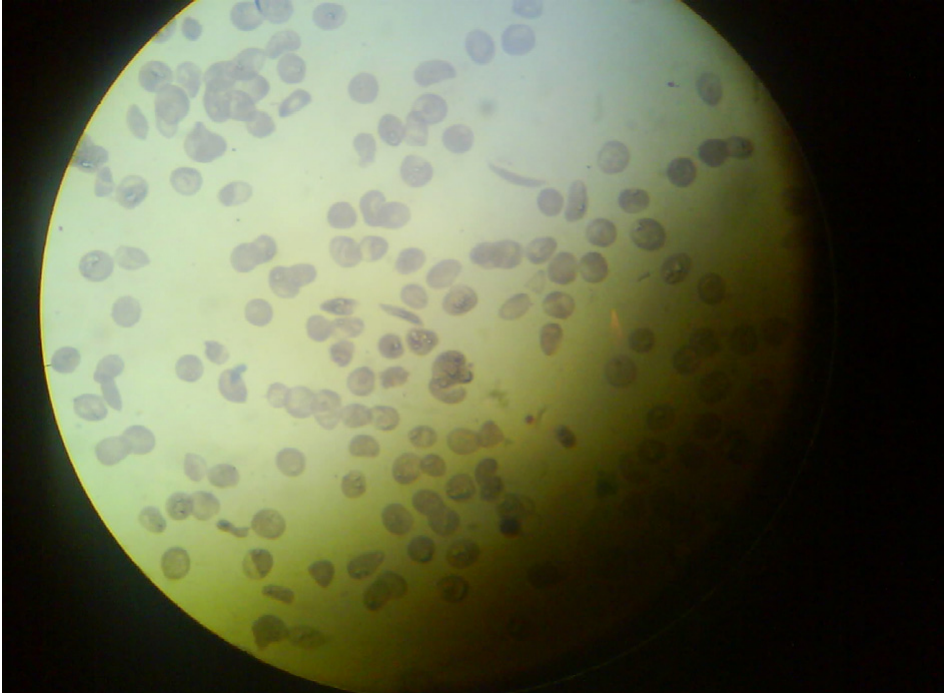
Prepheral blood smears with sickle cell.

In follow up of Hb electrophoresis, Hb s was 80%, Hb F: 18%, and Hb A2: 2%. Finally in peripheral blood smear, sickling of RBC was detected and the patient was diagnosed with sickle cell anemia and acute splenic sequestration crisis which was associated with acute chest syndrome treated with wide spectrum antibiotic (cefotaxim and erythromycin) and transfusion exchange (Figure 3). The patient was discharged with stable clinical state after 8 days.

The clinical presentation of our patient is rare and unusual for initial presentation of sickle cell anemia. Also association between acute chest syndrome and splenic sequestration is unusual.
